# The Evolution of Finger Counting Strategies in Addition: A Three‐Year Longitudinal Study in Young Children

**DOI:** 10.1111/desc.70293

**Published:** 2026-10-07

**Authors:** Marie Krenger, Jeanne Bagnoud, Catherine Thevenot

**Affiliations:** ^1^ Institute of Psychology University of Lausanne Lausanne Switzerland; ^2^ Department of Psychology University of Hong Kong Hong Kong SAR China

**Keywords:** addition, arithmetic, calculation strategies, finger counting, longitudinal design

## Abstract

In this study, the developmental trajectory of children's finger‐based strategies for addition is described for the first time using a longitudinal design involving 207 children aged 4 ½ at the first testing point. Children were observed individually twice a year over a three‐year period. The trajectory typically followed by children corresponds to the one theoretically described in the literature as supporting the gradual internalization of finger strategies and leading to the complete mentalization of the solving process. More precisely, children progressed from a modeling strategy in which each operand of an addition is represented on the fingers of each hand, through a concrete counting‐on strategy, in which both operands are still represented but enumerated in a single continuous process, to a counting‐on strategy, in which only one operand is kept in mind while the other is tracked on fingers. The frequent use of the concrete counting‐on strategy between modeling and counting‐on strategies supports Baroody's proposition that it serves as a transitional step between concrete and more abstract forms of numerical representation. However, the concrete counting‐on strategy was associated with poor addition performance, suggesting that it corresponds to a reconstructive stage in which children gain new insights about numbers but lack the cognitive resources to fully capitalize on them. Overall, this study provides the first detailed description of the evolution of finger‐based strategies toward the internalization of the addition solving process.

## Summary

- Using a longitudinal design, this study provides the first detailed description of children's developmental sequence of finger‐counting strategies in addition.
- Findings show that children initially model addition problems on their fingers and typically progress toward a counting‐on strategy before achieving full mental calculation.
- A transitional strategy bridging concrete and abstract approaches is also identified.
- This strategy involves enumerating both operands in a single, continuous counting process from 1 to the final sum

## 1 Introduction

Children frequently count on their fingers to solve addition problems (Butterworth 1999; Krenger and Thevenot 2026; see Neveu et al. 2023 for a review). Regrettably, and despite the fact that several studies indicate that young children who use their fingers to solve additions perform better than those who do not (Dupont‐Boime and Thevenot 2018; Jordan et al. 2008; Krenger and Thevenot 2024; Krenger et al. 2027; Poletti et al. 2025), teachers in kindergarten and primary school still often associate finger counting with math difficulties (Mutlu et al. 2020; Poletti et al. 2023). This negative view of finger counting may stem from the observations that, by the age of 7 or 8, children who do not count on their fingers outperform their peers who do (Jordan et al. 2008). However, recent evidence revealed that efficient non‐finger users at this age are in fact former finger users who have already internalized the strategy (Krenger and Thevenot 2026). This result is compatible with the idea that early finger‐based strategies support the development toward efficient mental strategies (Andres et al. 2008; Fayol and Seron 2005; Krenger and Thevenot 2025; Poletti et al. 2022; Thevenot et al. 2025).

A theoretical account of how the internalization of finger‐based strategies could be supported by finger use itself has been provided by Baroody (1987). This researcher hypothesized a developmental continuity from initial concrete strategies, in which fingers directly represent quantities, to more abstract strategies, in which fingers serve as a mean of keeping track of the addition process to determine a sum (Fuson 1982). More precisely, at the earliest developmental stage, children rely on modeling strategies in which both operands are represented on fingers. The sum is then obtained by counting all raised fingers. For example, to solve 3 + 2, a child might raise three fingers for the first operand and two fingers for the second, then count “1, 2, 3, 4, 5.” This strategy, often referred to as the counting‐all strategy (e.g., Fuson 1982), will be labeled the “modeling” strategy here (Carpenter and Moser 1982). Over time, building on this strategy, children may adopt a more abstract, continuous counting process, in which the two operands are enumerated in a single, uninterrupted sequence. At this stage, children may enumerate the two operands starting from one (i.e., 1, 2, 3, 4, 5 in our example). This strategy was qualified by Baroody as a modified counting‐all strategy. According to Steffe et al. (1982), before children can implement this continuous single process, they cannot conceive the sum as the extension of one set beyond another prior to initiating a solution. From this intermediate stage, children could gradually shift toward strategies in which they no longer need to represent both operands. Instead, they could initiate counting directly from one of the operands in the problem. The addition 3 + 2 could therefore be solved by starting from “3” and continuing “4, 5,” while raising only two fingers.

Although this gradual transition from concrete to more abstract strategies has been theoretically conceptualized, empirical evidence for this developmental pattern at a large scale is still lacking. Indeed, to date, children's finger‐based strategies have only been observed in small samples (Baroody 1987; Carpenter and Moser 1984; Fuson 1988) and have never been examined longitudinally. The present study aims to address these gaps by documenting the strategies children use from ages 4 ½ to 7 ½ to solve simple additions. Special attention will be given to the description of emerging finger‐based strategies and to the question of whether the development of more advanced strategies is systematically preceded by more basic ones and consistently leads directly to mentalization.

Examining the progression of children from finger modeling strategies to mental strategies is particularly interesting because it reflects children's gradual understanding of the addition concept (Fuson 1982). As illustrated by Siegler's overlapping waves model (1996), children may not transition from one strategy to another in a sudden and definite manner. Even after the emergence of a new strategy based on a better conceptual understanding of addition, this understanding can remain fragile and dependent on the problems’ surface characteristics (Piaget and Inhelder 1964/1941), such as the size of the numbers involved (Ollivier and Lemaire 2024). For example, a child might use a modified counting‐all strategy for 5 + 4, representing the numbers 1 to 9 on fingers and counting them in a single continuous sequence, but be reluctant to use the same strategy for 6 + 3, because one of the operands exceeds the number of fingers on one hand, or for 7 + 4, because it exceeds what can be represented on two hands. This would suggest that the modified counting‐all strategy remains tied to specific situations and that the abstract, general concept on which it depends, namely the extension principle described above (Steffe et al. 1982), has not yet been fully constructed.

To date, only two studies have examined how problem characteristics can influence the type of finger strategies children use. In one study, Dupont‐Boime and Thevenot (2018) reported that six‐year‐old children with higher working memory capacity are more likely to use a counting‐on strategy than their peers with lower capacity, particularly for additions with sums greater than 10. In contrast, children with lower working memory capacities more often rely on modeling strategies, which enables reasonable success for additions up to 10 but led to poorer performance when sums exceeded 10. These findings highlight the importance of both individual differences and problem characteristics in shaping strategy use and performance. However, this study offers limited insight into developmental trajectories, as it does not address how strategies evolve in relation to one another over time. Moreover, the authors’ focus was restricted to modeling and counting‐on strategies, without considering potential transitional strategies (i.e., modified counting all) that may mediate the gradual internalization of addition procedures (Baroody 1987). Finally, the binary distinction between sums below versus above 10 may obscure more subtle ways in which children adapt their strategies to task demands. For instance, as evoked above, additions with sums under 10 can still be challenging to model when one operand exceeds 5 because it prevents children from representing both operands separately on two hands. To capture the interaction between problem characteristics and finger‐based strategies over time, a more fine‐grained approach to the task material is therefore needed.

In the second study addressing the characteristics of problems affecting finger strategies, Bagnoud et al. (2025) used a longitudinal approach in 6‐year‐old children tested twice over a five‐month interval and observed that the developmental trajectories of strategy use diverged between tie, constructed with repeated operands, and non‐tie problems. Specifically, while children tended to shift directly from finger modeling to mental strategies for tie problems, a transitional step involving a counting‐on strategy was more frequently observed for non‐tie problems. While the developmental focus of this study is commendable, this study shares a limitation with Dupont‐Boime and Thevenot (2018) in that the description of strategies was restricted to the representation of one or two operands on fingers, without acknowledging the broader range of strategies children may use. Furthermore, the study did not consider operand size, a factor that can significantly influence children's ability to represent and manipulate quantities using their fingers.

The present study aims to overcome these shortcomings through longitudinal observations of finger‐based strategies, using addition sets carefully designed to elicit a range of different strategies. The three finger strategies described above will be considered, that is the modeling, the counting‐on strategies and the intermediate strategies consisting in representing the two operands in a single and continuous counting process. Instead of using the term “modified counting‐all” proposed by Baroody (1987), we chose to label this strategy “concrete counting‐on.” We did so because, in our view, this terminology better highlights its potential transitional role between a modeling strategy, in which the operands are concretely represented, and a counting‐on strategy, also characterized by a continuous enumeration process.

The use of these strategies was observed in 207 children aged 4½ during an addition task at the very beginning of their schooling (start of the Swiss education system, equivalent to prekindergarten). Observations continued until the beginning of their fourth year of school, when they turned 7½ (equivalent to Grade 2), with testing conducted every six months, resulting in a total of seven testing points. The addition task presented to children consisted in two sets of problems. Problems in set 1 were designed to allow easy implementation of a modeling strategy in which each operand can be separately represented on each hand (i.e., neither operand exceeding 5: 1 + 1, 2 + 2, 3 + 3, 4 + 4, 5 + 5, 1 + 2, 2 + 3, 3 + 4, 4 + 5, 1 + 5).). In contrast, this strategy could not be applied as easily to the problems in set 2, where the second operand was always larger than 5 and the sum exceeded 10 for three of the problems (i.e., 1 + 9, 2 + 8, 3 + 7, 4 + 6, 2 + 6, 3 + 8, 4 + 9, and 5 + 7). Children had to solve at least 8 out of 10 problems correctly in set 1 to proceed to set 2. Children were classified in skill level 1 when they were presented only with set 1, and in skill level 2 when they received both sets 1 and 2.

Concretely, for each addition, it was determined whether children used their fingers or not during the solving process. When fingers were used, it was determined whether a modeling, a concrete counting‐on, a counting‐on or a mental strategy was implemented. For each testing point, the frequency use of each strategy on the whole set(s) of problems presented was calculated. Whether children tended to rely on a single strategy at a given point or used multiple strategies within the same testing point could then be determined. These observations were complemented by considering children's addition skill level. Because the data exploited here have already been used in Krenger and Thevenot (2025) for a different purpose (with no mention of strategies), we already know that children who use their fingers at early developmental stages outperform those who do not. Consequently, we expect that finger users more often pass set 1 than non‐finger users. Over time, all children are expected to pass set 1 and therefore to reach skill level 2. More importantly, among finger‐based strategies, we expect young children to most frequently use the modeling strategy (Baroody 1987; Carpenter and Moser 1982; Dupont‐Boime and Thevenot 2018). Children using this strategy should be able to solve problems in set 1. However, they might be challenged by problems in set 2, in which, as previously noted, one of the operands exceeds 5. Therefore, the key question is whether and when children who rely on the modeling strategy will be able to implement a new finger‐counting strategy to overcome difficulties in set 2. For example, the concrete counting‐on strategy could allow children to solve not only set 1 but also set 2 problems. The counting‐on strategy is expected to emerge later during development (Baroody 1987; Fuson 1982). The next key question is whether, as predicted by Baroody's (1987) model, this counting‐on strategy is frequently preceded by a concrete counting‐on. More generally, the longitudinal design adopted here makes it possible to examine children's developmental trajectories by describing strategy distribution across testing points before the addition process is internalized, or, in other words, before exclusive reliance on mental strategies.

## 2 Method

### 2.1 Participants

A total of 211 children (108 girls) participated in the study. This sample size was determined by the number of parental consents obtained. Children were recruited from schools located in three relatively small cities in the Vaud Canton, Switzerland. They were predominantly white from middle to relatively high socioeconomic backgrounds. They were first observed at the beginning of their first year of school (prekindergarten; *Mage* = 4 years, 7 months, *SD* = 4 months) and tested every six months until the start of their fourth year (Grade 2; *Mage* = 7 years, 7 months, *SD* = 4 months). At the first testing point, data from 15 children who did not speak French well enough to understand the tasks were excluded. Their data were included in the analyses once their French proficiency was deemed sufficient. Nonetheless, the entire set of data from one child who was not proficient at T1 and T2 and moved away at T3, and from one child who was not fluent enough until the end of the study, were never considered. In addition, two children diagnosed with autism spectrum disorder participated only in a playful manner and their data were also excluded. This resulted in an initial sample of 207 children. However, a few technical issues with video recordings prevented data collection at specific testing points. The precise sample size, along with age and grade at each testing point, is presented in Table 1. It should be noted that most analyses were conducted using all available data, with only the missing observations at the affected testing points removed. The data from an entire child were removed only for the analyses of strategy developmental pathways, which were conducted on the 152 children who participated from the beginning to the end of the study during all sessions. Importantly, Little's (1988) MCAR test yielded a nonsignificant result, *χ^2^* (24) = 31.77, *p* = 0.130, providing no evidence that the pattern of missingness was systematically related to children's task performance or to other measured characteristics, such as finger use, gender, or age, or to specific additions or sums. Thus, the observed pattern of missing data was compatible with the assumption that data were missing completely at random (MCAR).

**TABLE 1:** School grade and sample description (number of girls, exclusion details, mean age (year; month), mean age (month) and standard deviation, maximum and minimum age) at each testing point.

		T1	T2	T3	T4	T5	T6	T7
Testing point		Prekindergarten		Kindergarten		Grade 1		Grade 2
Grade		Beginning	End	Beginning	End	Beginning	End	Beginning
Sample size *(N)*		192	192	189	184	165	166	165
*N* girls		96	100	99	95	91	89	90
*N* of exclusion		15	15	18	23	42	41	42
Limited French proficiency		13	9	1				
Missing data		2	3	2	2	5	1	
Have moved out			3	15	21	37	40	42
Age								
Year; Month	Mean	4;7	5;1	5;7	6;2	6;7	7;1	7;7
Month	Mean	55.63	60.87	66.73	74.37	79.04	84.74	91.33
sd		3.77	3.77	3.80	3.76	3.82	3.79	3.73
min		46	51	57	65	70	75	82
max		62	68	74	82	86	92	98

*Note*: Two children with ASD and two children whose French proficiency was insufficient at all testing points (one of whom moved away at T3) are not included in this table because their data were never used.

Although children were recruited from different schools in the Vaud Canton, all schools are required to follow the French speaking Swiss curriculum(Plan d'Etude Romand, PER), which defines the learning objectives and methods for each school year and ensures substantial curricular harmonization across schools. Regarding mathematics instruction, in Sessions T1, T2, T3 and T4 in our design, the PERspecifies that children should work on arithmetic problems involving numbers from 1 to 12, while becoming familiar with numbers up to 30. In Grade 1 , arithmetic problems extend to numbers up to 20, and in Grade 2 up to 200. Concerning addition strategies, the PER indicates that children are initially taught to solve additions using counting procedures during Grade 1. From Grade 2 onward, instruction expands to include more advanced strategies such as counting‐on using number lines or number charts, retrieval, and decomposition strategies. The curriculum also specifies that children should progressively master the basic addition facts (from 0 + 0 to 9 + 9) and retrieve them from memory. Importantly, the PER does not provide explicit recommendations either encouraging or discouraging finger‐based calculation. Thus, finger use is not formally addressed in the official curriculum.

The study received ethical approval from the Ethics Committee of the Faculty of Social and Political Sciences (SSP) of the University of Lausanne .

### 2.2 Addition Task

In the addition task, children had to solve additions presented individually in Arabic numbers on A5‐sized paper cards and to provide their answers orally. No time limit was imposed, and no specific instructions were given regarding the use of fingers to solve the additions. If children requested or the experimenter noticed discrete finger use, the experimenter confirmed that they were free to use their fingers as they wanted. Given children's young age and possible difficulties reading symbolic numbers at the first testing points, the experimenter read the additions aloud unless a child began solving the problem immediately after card presentation. When children said that they did not know the answer, the experimenter encouraged them to try to solve it by saying: “How could you know the answer?”

The addition task included four sets of additions of increasing difficulty, but only the first two sets were used in the present study. The first set was administered to all children, whereas the second set was presented only to those who achieved at least 80% correct responses on the first set. More specifically, all children were presented with the first set, which consisted of 10 additions with both operands ranging from 1 to 5. All five tie additions within this range were included (i.e., 1 + 1, 2 + 2, 3 + 3, 4 + 4, and 5 + 5). For the non‐tie additions, each digit appeared twice across problems, and the smaller operand was always presented first (i.e., 1 + 2, 2 + 3, 3 + 4, 4 + 5, 1 + 5). Only children who correctly solved at least 8 out of 10 additions were presented with the second set. This second set consisted of 8 additions in which the first operand ranged from 1 to 5 and the second operand from 6 to 9, yielding sums between 8 and 13 (sums could potentially reach 14, but were limited to a maximum of 13 by our selection). All complements to 10 within this range were included (i.e., 1 + 9, 2 + 8, 3 + 7, and 4 + 6), and the remaining additions were constructed so that each operand appeared the same number of times (i.e., 2 + 6, 3 + 8, 4 + 9, and 5 + 7). Children were considered to be at skill level 1 if they were only presented with set 1 and failed to meet the performance criterion for set 2, and at skill level 2 if they were presented with set 2.

Children's behavior during the task was videorecorded to retrospectively analyze the strategies they used to solve the additions and ensure reliable coding. Children's answers and use of fingers were coded using the software BORIS (v. 7.4.11 – 2019–02‐28; Friard and Gamba 2016). Children were considered finger users if, at least once while solving an addition in sets 1 or 2, they visibly moved their fingers sequentially, represented quantities by raising fingers, or physically touched their fingers for counting. In contrast, children who did not exhibit any of these behaviors were classified as non‐finger users.

Then, for the addition solved with fingers, the precise strategy children used was identified. The “modeling” strategy was coded when children first represented both operands on their fingers and then determined the sum by counting all raised fingers. The “concrete counting‐on” strategy was coded when both operands were counted one after the other, in a counting on and continuous process. Finally, the “counting‐on” strategy was coded when only one operand was represented on fingers and counted from the other operand. Whenever children's gestures were too subtle or did not match any defined strategy, pivotal verbal cues were absent, or they hid their fingers, the strategy could not be classified and was assigned to the “Other” category.

To ensure the reliability of our analyses, an independent rater was asked to code 1204 observations (6.35% of our observations, from 68 different children and/or testing point). The interrater agreement was 98% on the identification of whether the addition was solved with fingers (*k* = 0.962, *z* = 33.39, *p* <0.001), indicating a high level of coding reliability. For the coding of the precise strategies used by children, the interrater agreement was 95% (*k* = 0.898, *z* = 48.271, *p* <0.001), once again indicating a high level of reliability. The few disagreements between raters were resolved through discussion.

Our coding at each testing point identified which strategies (modeling, concrete counting‐on, or counting‐on) each child used at least once, revealing the predominant strategies when they started and just before they stopped using fingers, and allowing us to trace their strategy development from T1 to T7. To provide a clear overview of the variety of strategy combinations across sessions, we simplified them by removing consecutive repetitions of the same combinations, by ignoring sessions with only mental strategies, and by disregarding “other” finger strategies. For example, the following sequence: T1 mental only, T2 other only, T3 modeling and other, T4 concrete counting‐on, T5 concrete counting‐on and counting‐on, T6 counting‐on, T7 mental only, was simplified to:modeling → concrete counting‐on → concrete counting‐on and counting‐on → counting‐on.

### 2.3 Statistical Analysis

Chi‐squared tests were used to assess the occurrence of finger use across different testing points. Mixed‐effect binomial models were used to determine the occurrence of strategies across testing points, factoring children's skill levels and/or the set of problems, depending on the specific analysis. To account for repeated measurements on the same children, a random intercept was included in the models. The function glmer of the library lmerTest (Kuznetsova et al. 2017) was used to construct the models. Marginal (R2m: variance explained by the fixed effects only) and conditional R‐squared (R2c: variance explained by both fixed and random effects) were calculated with the MuMIn package (Barton 2023), and the delta method was reported because it provides a more general approximation. In order to test the significance of predictors and their interactions, a Type II Wald chi‐square Analysis of Deviance table was generated with the Anova function of the car package (Fox and Weisberg 2019). Contrasts were calculated with the emmeans package (Lenth 2025). For the sake of concision and simplicity, nonsignificant results are reported only when theoretically relevant. Moreover, only sequential contrasts of interest are reported. However, all other possible pairwise comparisons were performed, but they are reported only when they potentially contradicted the overall result pattern. Contrasts are reported when interactions are significant, except for interactions involving the seven testing points, where only a general description is provided due to the complexity of detailing all contrasts. Finally, to provide a more realistic representation of our results, the percentages reported in the text and tables are based on raw data, calculated by directly averaging over items, rather than on estimated marginal means, which were used in the linear model analyses.

The first section of the results examines whether children used each strategy at least once, without considering frequency. The second section considers strategy frequency, analyzing each problem individually in an item‐based format. Strategy analyses were conducted regardless of whether problems were solved correctly. For accuracy, mixed‐effects binomial models were also applied at the item level, using the same approach as for the strategy occurrence analyses.

### 2.4 Transparency and Openness Statements

The study design and analyses were not preregistered. The analyses reported here are entirely new, although they rely on the same dataset as a previously published manuscript (Krenger and Thevenot, 2026). Only two descriptive elements mentioned here were previously reported in the earlier paper: (1) the number of finger users at each testing point, and (2) a descriptive report of the percentages of the different strategies used at each testing point. Importantly, this last information was included only for descriptive purpose and was not accompanied by analyses, interpretation, or discussion. Thus, the present paper is the first to provide an analysis of children's finger strategies. The dataset Krenger and Thevenot (2025) is openly available at:https://osf.io/543yr/overview?view_only=5937fa7b3412495f9849e60c629c1368.

## 3 Results

### 3.1 Strategy Use at the Child Level

#### 3.1.1 Numbers of Finger Users and Non‐Finger Users in Skill Level 1 and 2 Across Testing Points

The numbers of finger users and non‐finger users in skill level 1 or 2 are presented in Table 2. As a reminder, children were classified as belonging to skill level 1 when they did not reach the success criterion in set 1 (i.e. 80% of additions solved correctly) and were therefore not presented with set 2. Conversely, they were classified as belonging to skill level 2 when they passed set 1 and were presented with set 2. A series of *χ^2^* tests indicated that the proportions of level 2 children was significantly higher among finger users than non‐finger users up until T5 when children were aged 6 ½ (*χ^2^* (1, *N* = 192) = 30.06, *p* < 0.001 at T1; *χ^2^* (1, *N* = 192) = 41.65, *p* < 0.001 at T2; *χ^2^* (1, *N* = 189) = 65.18, *p* < 0.001 at T3; *χ^2^* (1, *N* = 184) = 31.47, *p* < 0.001 at T4; *χ^2^* (1, *N* = 165) = 18.08, *p* < 0.001 at T5). At T6 and T7, at the age of 7 and 7 ½, almost all children, finger users or not, were skill level 2 children, which necessarily resulted in nonsignificant differences in the distribution of children between levels depending on finger use (*χ^2^* (1, *N* = 166) = 0.23, *p* = 0.63 at T6; *χ^2^* (1, *N* = 165) = 0.0003, *p* = 0.99 at T7).

**TABLE 2:** Numbers of skill level 1 and 2 children depending on their use of fingers in the addition task across testing points.

	T1	T2	T3	T4	T5	T6	T7
	4½ y‐o	5 y‐o	5½ y‐o	6 y‐o	6½ y‐o	7 y‐o	7½ y‐o
Skill level 1	180	158	119	56	34	5	1
Finger users	24	24	21	17	12	3	0
Non‐finger users	156	134	98	39	22	2	1
Skill level 2	12	34	70	128	131	161	164
Finger users	9	23	54	95	97	79	81
Non‐finger users	3	11	16	33	34	82	83
Total	192	192	189	184	165	166	165

#### 3.1.2 Numbers of Finger Users Using the Different Finger‐Based Strategies (Modeling, Concrete Counting‐on, and Counting‐on Strategies)

The numbers of children using different strategies across testing points are shown in Table 3. A mixed‐effects binomial model was fitted with the Type of Strategy Users (Modeling, Concrete Counting‐on, and Counting‐On Strategies) and Testing Points (T1–T7) as predictors of strategy use at the child level (R2m: 0.15, R2c: 0.15). The effect of the Type of Strategy Users was significant, *χ^2^* (2, *N* = 1617) = 45.72, *p* < 0.001, showing that children using the modeling strategy were more numerous than children using the concrete counting‐on or the counting‐on strategies (see Table 3). This effect interacted with Testing Points, χ^2^(2, *N* = 1617) = 116.35, *p* < 0.001, showing that, whereas the modeling strategy was associated with the highest number of children until T5, counting‐on users who were initially underrepresented until T4, became dominant at T7 (Table 3).

**TABLE 3:** Numbers of children (N) using the different finger‐based strategies and mean number of strategies across testing points.

	T1	T2	T3	T4	T5	T6	T7
Children using only one strategy	21	31	46	58	64	56	62
Modeling	10	15	33	43	38	19	11
Concrete counting‐on	10	12	8	7	4	8	2
Counting‐on	1	4	5	8	22	29	49
Other	6	4	10	11	7	4	7
Children using two strategies	6	11	17	40	34	19	9
Modeling and concrete counting‐on	6	9	14	25	24	10	2
Modeling and counting‐on	—	1	1	3	5	5	3
Concrete counting‐on and counting‐on	—	1	2	12	5	4	4
Children using the three strategies	—	1	2	3	4	3	3
N total using the modeling strategy	16	26	50	74	71	37	19
N total using the concrete counting‐on	16	23	26	47	37	25	11
N total children using the counting‐on strategy	1	7	10	26	36	41	59
N total finger users	33	47	75	112	109	82	81
Mean N of strategies used	1.22	1.19	1.32	1.46	1.41	1.32	1.20

#### 3.1.3 Numbers of Children Using each Strategy Combination at Their First and Last Finger‐Use Session

Among all children included in this study, 152 were tested from the first to the last testing point. Among them, 10 never used their fingers (see Krenger and Thevenot 2026 for details on these children). For the remaining 142 children, the strategies used at their first observed session of finger use are reported in Table 4. At this first point of finger use, 62 children (43.7%) used a modeling strategy only, 20 (14.1%) used a concrete counting‐on strategy only, and 27 (19.0%) used both strategies. Only 7 (4.9%) used a counting‐on strategy only. To examine how long children continued to rely on their fingers depending on their initial strategy combination, we calculated the mean number of sessions in which finger use was observed^1^. Children who started with the concrete counting‐on strategy relied on their fingers the longest, averaging 4.6 sessions. In contrast, those who began with the counting‐on strategy used their fingers for an average of 2.1 sessions. Then, we examined which strategies children used immediately before addition was internalized. This was done in the 70 children (out of 142) who had stopped using their fingers before the end of our observations. Just before internalization, 28 of them used a modeling strategy, 14 used a concrete counting‐on strategy, and 31 used a counting‐on strategy. The total number of reported strategies exceeds 70 because some children used more than one strategy before internalization (see Table 4).

**TABLE 4:** Numbers of children (N) (*and %*) who used the different finger‐based strategies at their first and last testing point of finger use, and mean numbers of sessions of finger use.

	*N* of children using these strategies at first (*N* = 142)			*N* of children using these strategies at last (*N* = 70)	
Strategy combination	N	%	Mean *n* of sessions of finger use	N	%
One strategy					
Modeling	62	43.7	3.2	18	25.7
Concrete counting‐on	20	14.1	4.6	2	2.9
Counting‐on	7	4.9	2.1	26	37.2
Other only	18	12.7	2.6	11	15.7
Two strategies					
Modeling and concrete counting‐on	27	19.0	3.6	8	11.4
Modeling and counting‐on	0	0	—	1	1.4
Concrete counting‐on and counting‐on	7	4.9	3.1	3	4.3
Three strategies	1	0.7	3.0	1	1.4
Total	142	100	3.3	70	100

***Note***: “Mean *n* of sessions of finger use” corresponds to the mean number of sessions in which children were observed using their fingers.

The number of children who engaged in different developmental pathways of strategy combination is reported in Appendix A. Note that, as explained in the Method section, these developmental trajectories do not take into account the “other” finger strategy, nor the repetition of the same strategy combination between two consecutive sessions, and sessions with only mental strategy. Among the 142 children, 57 (40%) used all three strategies during the course of their development (i.e., modeling, concrete counting‐on, and counting‐on), 54 (38%) used two strategies, and 31 (22%) used only one of the three strategies. Among the 57 children who used all three strategies, 40 showed trajectories where the concrete counting‐on emerges either after or with the modeling strategy and before or with the counting‐on strategy. More precisely, 13 children progressed from modeling to concrete counting‐on and then to counting‐on, 4 began with modeling before using the two other strategies together, 21 discovered modeling and concrete counting‐on at the same time before the counting‐on, and 2 discovered all three strategies simultaneously. However, for the 17 other children, concrete counting‐on emerge either before the modeling strategy or after the counting‐on. More precisely, for 11 of them it appeared before both modeling and counting‐on, for 4 it emerged together with counting‐on before modeling, in 1 counting‐on came first followed by the two other strategies, and in 1 modeling was followed by counting‐on and finally by concrete counting‐on. Among the 54 children who used two strategies, 45 followed an order consistent with gradual internalization, meaning either modeling preceding concrete counting‐on (*n* = 9), modeling preceding counting‐on (*n* = 18), concrete counting‐on preceding counting‐on (*n* = 5), or the simultaneous discovery of two strategies (*n* = 13, 8 children for modeling and concrete counting‐on, 2 children for modeling and counting‐on, and 3 children for concrete counting‐on and counting‐on). Only 9 children followed a reverse order, with 7 beginning with concrete counting‐on before modeling, and 3 beginning with the counting‐on before the modeling. Finally, among the 31 children who used only one single strategy, 17 relied exclusively on modeling, 7 relied exclusively on counting‐on (and 7 relied only on “other” strategies).

### 3.2 Frequency of Strategy Use by Finger Users

#### 3.2.1 Overall Strategy Use (Modeling, Concrete Counting‐On, Counting‐On, and Mental Strategies) by Finger Users

Among finger users, the percentages of use and the percentages of correct responses of the different finger‐based strategies and the mental strategy are presented in Table 5. A mixed‐effects binomial model was fitted with the Strategy (Modeling, Concrete Counting‐on, Counting‐On, and Mental Strategies) and Testing Points (T1–T7) as predictors of strategy use at the item level (R2m: 0.30, R2c: 0.30). An analysis of deviance on this model revealed no significant main effects of Testing Point, χ^2^(1, *N* = 33256) = 0.30, *p* = 0.58. The main effect of the Strategy was significant, χ^2^(3, *N* = 33256) = 6651.37, *p* < 0.001. Contrasts showed that, overall, mental strategies were more often used than modeling ones (*z* = 56.85, *p* < 0.001), which were in turn more frequent than the concrete counting‐on strategy (*z* = 20.22, *p* < 0.001), in turn more frequent than the counting‐on strategy (*z* = 4.70, *p* < 0.001). The interaction between Strategy and Testing Points was significant, χ^2^(3, *N* = 33256) = 1142.68, *p* < 0.001. Contrasts indicated that there was a decrease in the frequency of concrete counting‐on (*z* = 16.00, *p* < 0.001) and modeling strategies (*z* = 19.03, *p* < 0.001) over time, while there was an increase in the frequency of counting‐on (*z* = ‐11.94, *p* < 0.001) and mental strategies (*z* = ‐19.50, *p* < 0.001; see Table 5).

**TABLE 5:** Percentages of strategies used and percentages of correct responses (*acc*.) at each testing point in finger users.

	T1		T2		T3		T4		T5		T6		T7	
Strategy	use	acc.	use	acc.	use	acc.	use	acc.	use	acc.	use	acc.	use	acc.
Modeling	21	64	28	57	29	58	25	67	20	73	10	79	4	82
Concrete counting‐on	12	42	17	45	13	50	10	55	7	47	4	70	1	79
Counting‐on	1	100	2	100	2	79	4	89	5	88	8	85	12	92
Other	15	15	8	33	8	45	6	62	6	75	6	82	3	89
Mental	51	38	46	59	49	72	54	80	62	85	72	94	80	96

A more detailed account of the different finger‐based strategies used by children depending on the characteristics of the problems can be found in Appendix B, noticeably concerning tie and non‐tie problems for Set 1 and complement to 10 or not for Set 2.

#### 3.2.2 Strategy Use (Modeling, Concrete Counting‐on, Counting‐on, and Mental Strategies) by Finger Users According to Their Skill Level and Set of Problems

The percentages of use and of correct responses of each strategy, depending on their use in set 1 and set 2 across the seven testing points for skill level 1 and 2 children, are illustrated in Figure 1 and precisely reported in Appendix C. The frequencies of strategies used have been converted into percentages for easier reading. As for the preceding result section, the present analyses were conducted independently of problem accuracy. Two different mixed‐effects binomial models were fitted to determine the frequency of strategy use. The first one was conducted for set 1 only with Strategy (Modeling, Concrete Counting‐on, Counting‐On, and Mental Strategies), Level (Level 1, Level 2) and Testing points (T1 to T7) as predictors (R2m: 0.45, R2c: 0.45). The second one was conducted for skill level 2 children only and the model was constructed with Strategy (Modeling, Concrete Counting‐on, Counting‐On, and Mental Strategies), Set (Set 1, Set 2), and Testing points (T1 to T7) as predictors (R2m: 0.37, R2c: 0.37). Results of the analysis of deviance, the contrasts, and the estimated marginal means are presented separately for the two analyses.

**FIGURE 1:**
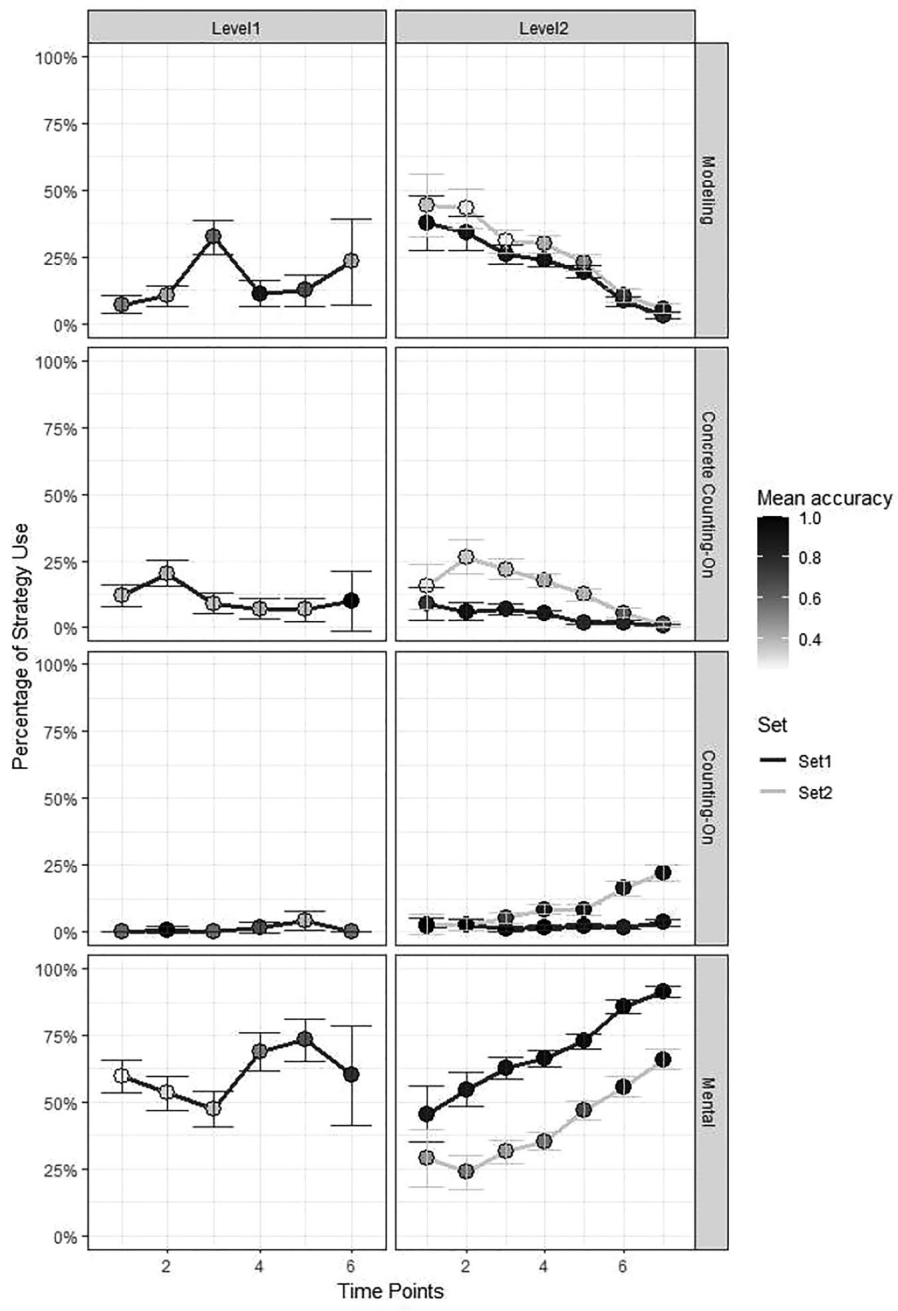
Percentages of strategy use (modeling, concrete counting‐on, counting‐on, and mental strategies) and percentages of correct answers across testing points on set 1 for skill level 1 finger users and on set 1 and 2 for skill level 2 finger users. *Note*: The error bar corresponds to the confidence interval of the strategy use, set at 95%.

An analysis of deviance on the model on Set 1 revealed a significant effect of Strategy (71% of mental strategy, 17% of modeling strategy, 5% of concrete counting‐on and 2% of counting‐on), χ^2^(3, *N* = 20504) = 4761.62, *p* < 0.00. This effect was modulated by Testing Point, χ^2^(3, *N* = 20504) = 516.19, *p* < 0.001. Contrasts indicated that there was a decrease in the frequency of concrete counting‐on (*z* = 7.41, *p* < 0.001) and modeling strategies (*z* = 4.80, *p* < 0.001) over time, while there was an increase in frequency of counting‐on (*z* = ‐2.69, *p* = 0.02) and mental strategies (*z* = ‐8.13, *p* < 0.001). The interaction between Strategy and Level was also significant, χ^2^(3, *N* = 20504) = 70.00, *p* < 0.001, indicating that the concrete counting‐on strategy was more used by skill level 1 (12%) compare to skill level 2 children (3%; *z* = 5.17, *p* < 0.001), while mental strategy was more often used by skill level 2 (74%) than skill level 1 children (59%; *z* = ‐3.03, *p* = 0.003). The contrast showed no overall difference between the children's level for the counting‐on (1% vs. 2%, respectively for skill level 1 and 2 children, *z* = 0.13, *p* = 0.89) and modeling strategies (15% vs. 17%, respectively for skill level 1 and 2 children, *z* = 1.04, *p* = 0.30). This effect was however, modulated by the three‐way interaction between Strategy, Level, and Testing Points, χ^2^(3, *N* = 20504) = 124.35, *p* < 0.001, showing that at the first three testing points, skill level 2 children used the modeling strategies more frequently and the mental strategy less frequently compared to skill level 1 children, but that the reverse pattern was observed in the 3 last testing points (T5‐T7; see Figure 1). In contrast, skill level 1 children used the concrete counting‐on strategy more frequently than the skill level 2 children across all testing points. Finally, and as seen in Figure 1, the counting‐on strategy was rarely used, and its frequency was the same across testing points and skill level.

An analysis of deviance on the model on skill level 2 children revealed a significant effect of Strategy, χ^2^(3, *N* = 29760) = 4400.06, *p* < 0.001 and Set, χ^2^(1, *N* = 29760) = 4.39, *p* = 0.04, but also an interaction between these two variables, χ^2^(3, *N* = 29760) = 1059.21, *p* < 0.001. Contrasts indicated that there was a more frequent use of the mental strategies in Set 1 (74%) than in Set 2 (46%; *z* = 23.58, *p* < 0.001), while there was a less frequent use in Set 1 than in Set 2 of the concrete counting‐on (3% vs. 12%, for Set 1 and 2 respectively, *z* = ‐12.83, *p* < 0.001), the counting‐on (2% vs. 12%, for Set 1 and 2 respectively, *z* = ‐14.20, *p* < 0.001) and the modeling strategies (17% vs. 21%, for Set 1 and 2 respectively*, z* = ‐5.72, *p* < 0.001). The interaction between Strategy and Testing Points was significant, χ^2^(3, *N* = 29760) = 1343.70, *p* < 0.001 and showed that there was a decrease in the frequency of concrete counting‐on and modeling strategies over time, but an increase in the frequency of mental strategies and counting‐on. However, as attested by a Strategy x Testing Point x Set, χ^2^(3, *N* = 29760) = 14.34, *p* = 0.002, this increase in the counting‐on was only observed in Set 2, while for all the other strategies the effects just described were similar for both sets (Figure 1).

### 3.3 Percentages of Correct Responses Associated With Strategy Use (Modeling, Concrete Counting‐on, Counting‐on, and Mental Strategies) and the Set of Problems in Finger Users

As a reminder, Set 1 included additions with operands up to 5, allowing for a modeling strategy where each operand can be represented on each hand. Set 2 had additions with the first operand up to 5 and the second between 6 and 9, making the modeling strategy impractical due to the larger second operand. This could result in differences in accuracy depending on the strategy and the set. In order to study such effects, a mixed‐effects binomial model was fitted to examine the impact of Strategy (modeling, concrete counting‐on, counting‐on, and mental strategies), Testing Points (T1–T7), and Set (Set 1 and Set 2) on the percentages of correct responses (R2m: 0.34, R2c: 0.49). An analysis of deviance on the model revealed a significant main effect of Testing Point, χ^2^(1, *N* = 8314) = 765.99, *p* < 0.001, indicating that the percentages of correct responses increased with time. An effect of Set was detected, χ^2^(1, *N* = 8314) = 773.01, *p* < 0.001, with an overall higher percentage of correct responses in Set 1 compared to Set 2. Moreover, the interaction between Set and Testing Point was significant, χ^2^(1, *N* = 8314) = 8.50, *p* = 0.004. Contrasts indicated that the difference in the percentages of correct responses between the two sets reduced over time. The effect of Strategy was significant, χ^2^(3, *N* = 8314) = 25.22, *p* < 0.001, showing that the percentage of correct responses was lower for the concrete counting‐on strategies than for all the other strategies. The interaction between Set and Strategies was significant, χ^2^(3, *N* = 8314) = 27.70, *p* < 0.001. Even though the percentages of correct responses was significantly higher in Set 2 than Set 1 for each strategy, (Counting‐On: *z* = 2.76, *p* = 0.01; Concrete Counting‐On: *z* = 7.46, *p* < 0.001; Modeling: *z* = 15.72, *p* < 0.001; Mental: *z* = 19.53, *p* < 0.001), the difference between sets was higher for the modeling and the concrete counting‐on strategies than for the mental or the counting‐on strategies. The interaction between Strategy and Testing Points was significant, χ^2^(3, *N* = 8314) = 47.45, *p* < 0.001. As illustrated in Figure 2, even if there were a significant increase of the percentages of correct responses for all strategies (Counting‐On: *z* = ‐3.05, *p* = 0.01; Concrete Counting‐On: *z* = ‐10.05, *p* < 0.001; Modeling: *z* = ‐13.32, *p* < 0.001; Mental: *z* = ‐26.67, *p* < 0.001), the percentage of correct responses associated with mental strategies increased more than those associated with finger‐based strategies. Specifically, initially, the accuracy for mental strategies was lower than that of all finger‐based strategies, but it reached the highest percentages of correct responses of all strategies by the end. The three‐way interaction between Strategy, Set, and Testing Points was significant, *χ^2^* (3, *N* = 8314) = 13.74, *p* = 0.003. Contrasts indicated that for Set 1, there was no significant increase in the percentages of correct responses with time for the counting‐on strategy (*z* = ‐0.43, *p* = 0.90), but significant increase for modeling (*z* = ‐7.17, *p* < 0.001), concrete counting on (*z* = ‐6.76, *p* < 0.001), and for mental strategies (*z* = ‐23.43, *p* < 0.001) (see Figure 2), whereas for Set 2, the percentage of correct responses significantly increased with time for all strategies, and as seen in Figure 2, with noticeable increase for mental strategies.

**FIGURE 2:**
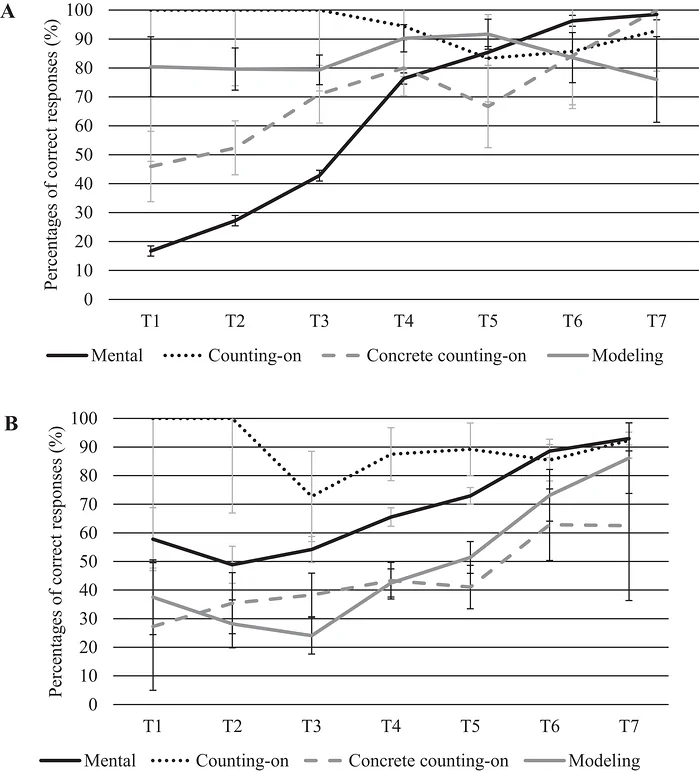
Frequency of correct responses on set 1 (A) and 2 (B) depending on the strategy and testing point. *Note*: The error bars correspond to the confidence interval set at 95%.

In a final analysis, we aimed at determining whether children's profiles at an early session point could predict their performance at the end of our observations (T7) on the whole set of presented problems (max 46 points). We chose T3 as the predictor point because it is the first point where a substantial number of children used their fingers (i.e., 40% of them in contrast to only 25% at T2). Results showed that children's performance at the final testing point (T7) differed significantly depending on their strategy profile at T3, *F*(4, 149) = 4.32, *η^2^p* = 0.104*, p* = 0.002. Non‐finger users obtained the lowest scores across all groups (*M* = 33.3 pts). Among finger users, children who relied exclusively on modeling strategies showed the lowest performance (*M* = 36.1 pts). In contrast, children who used exclusively the concrete counting‐on (*M* = 41.9 pts) or the counting‐on strategy (*M* = 41.2 pts) achieved the highest scores, followed by children combining concrete counting‐on and modeling strategies (*M* = 39.9 pts).

## 4 Discussion

In this research, we aimed to describe for the first time the evolution of finger‐based strategies across development. To this end, we adopted a longitudinal design where children aged from 4 ½ to 7 ½ were observed while solving addition problems of increasing difficulty across seven testing points. More precisely, we were interested in determining the successive emergence of the modeling, concrete counting‐on and counting‐on strategies, with the idea that the more concrete strategies could give rise to more abstract ones (Baroody 1987; Fuson 1982).

Our results show that, among finger strategies, the modeling strategy, consisting of representing both addends before combining them to determine the sum, was the most commonly used^2^ until the age of 6 ½. Moreover, this strategy was also the one that most commonly emerged first during development. Interestingly, this modeling strategy was more often used by young children with the highest arithmetical skill level, who predominantly used this strategy on both Set 1 (71%) and Set 2 problems (47%), where one of the operands exceed the capacity of fingers of one hand. Nonetheless, this attempt was not associated with good performance on Set 2, and it is only from the age of 7 that children begin to shift to the more abstract counting‐on strategy, thereby becoming able to successfully solve less modelizable problems. These observations are consistent with previous accounts in the literature assigning to the modeling strategy a foundational role in the emergence of the counting‐on strategy (Fuson 1982).

This counting‐on strategy was used increasingly over time and became dominant among finger strategies when children were aged 7 ½, that is only at the last point of our observations. At this point, 20% of the problems were still solved using fingers, and almost two‐thirds of them were solved using a counting‐on strategy. This strategy was always associated with very good performance in the addition task, but it must be noted that it was virtually absent in children until the age of 6 ½. In accordance with what was expected, the counting‐on strategy was the dominant strategy right before internalization, and the rare children whom we observed using this strategy at their first session of finger use reached mentalization in fewer sessions than other children. These observations are consistent with previous accounts in the literature considering the counting‐on strategy as reflecting a conception of number on the edge of the possible internalization of the whole calculation process (Fuson 1982). Note, however, that the modeling strategy was also still used by many children just before internalization. This finding is consistent with Bagnoud et al. (2025) conclusion that shifts from counting to mentalization depend on the characteristics of the problems. More specifically, modeling strategies are also frequently used for tie problems immediately before their results can be retrieved from long‐term memory, whereas for non‐tie problems, children tend to internalize the counting procedure directly through counting‐on (see Prado and Thevenot (2026) on the implications of these results within a theoretical developmental account).

If, as just shown, the modeling strategy constitutes the basic approach used by children at the beginning of learning, and the counting‐on strategy represents the last stage before internalization, the question now is whether our data support the existence of a transitional strategy between the two. As explained in the Introduction, the concrete counting‐on strategy has been hypothesized to serve this bridging function, creating a link between the strict modeling of numbers on fingers and a more abstract representation of one of these numbers emerging from a counting‐on process (Baroody 1987). This concrete counting‐on strategy was indeed frequently used by children, with only 51 children out of 142 never observed using it during our observations. Moreover, among the 57 children who used the three strategies during the course of our observations, the most frequent developmental pathway between strategies, observed in 40 children, was the one where the concrete counting‐on strategy emerged between the modeling (or simultaneously with it) and the counting‐on strategies (or simultaneously with it). Finally, compared with the modeling and the counting‐on strategies, the concrete counting‐on strategies was more rarely observed as immediately preceding complete internalization of the addition solving process. All in all, these results support the idea of a transitional role of the concrete counting‐on strategies toward the counting‐on strategy.

Nonetheless, the concrete counting‐on strategy was used more often by children with the lowest addition skills and was associated with poor performance on the addition task, regardless of children's skill level. This may seem surprising for a strategy that is supposed to guide children toward a more advanced level of development. One possible interpretation is that the concrete counting‐on strategy reflects a new conceptual understanding, namely that the quantities involved in an addition do not need to be systematically (re)modeled during the solution process (Steffe et al. 1982) but comes at a performance cost because it places greater demands on working memory than the modeling strategy. Indeed, in the modeling strategy, both operands are externally represented, and solving the problem simply involves counting what is visible. In contrast, during concrete counting‐on, the second operand must be mentally represented while the child simultaneously produces a verbal counting sequence that no longer corresponds to perceptual input. These two conflicting counting tasks, counting the count‐on words, place a substantial load on working memory. It could be only after repetitive failure that children develop a new, more efficient strategy extending their new conceptual understanding that numbers in an addition do not need to be modeled and that the first number provided in a problem corresponds “to the summary of its verbal enumeration [and can thus] be substituted for this enumeration” (p.69, Fuson 1982). The continuous counting process initially used by children in a concrete counting‐on strategy could therefore reach full efficiency only within the counting‐on strategy.

Thus far, we have discussed children who demonstrated all three strategies across our observations. Regarding the 54 children who used only two strategies across testing points, 45 of them followed a pathway consistent with the hypothesis of gradual internalization of the addition process (i.e., modeling before concrete counting‐on; modeling before counting‐on; or concrete counting‐on before counting‐on). Within this group, 20 children who did not transition through the concrete counting‐on strategy, instead shifting directly from modeling to counting‐on before mentalization, are also considered to conform to the hypothesized pathway. Indeed, according to Fuson (1982), children transition can spontaneously shift from one strategy to another when they realize that, in the modeling strategy, they unnecessarily recount the same objects or repeat the same words.

Until now, we have described the trajectories that children most often follow toward mentalization, which are congruent with our hypothesis. However, it should be noted that 26 of the 142 children who displayed at least two strategies did not follow this pattern. For example, within children who used only two strategies, 7 used the concrete counting‐on strategy before the modeling strategy, and 2 used counting‐on before modeling. Moreover, 31 children were observed using only one strategy before reaching mentalization, which does not conform either with the idea of a gradual internalization of strategies. Despite further investigation using external measures of arithmetical abilities, we found no evidence that these children exhibited lower skills than those who more closely followed the hypothesized pathways toward internalization^3^. Then, although we identified a dominant developmental pattern in the emergence of finger strategies, it is important to keep in mind that several different behavioral profiles can be observed, and, that, as in many areas of development, children's progress cannot be reduced to a single, uniform pathway. Moreover, even among children described here as following the hypothesized pathway toward mentalization, numerous shifts back and forth between strategies across sessions (see Appendix A) confirm that cognitive development does not always follow a monotonic progression toward a final state (Siegler 1996).

Still, as demonstrated in the present research, there is an order in the succession of finger strategies that children preferentially adopt before solving addition problems mentally. Modeling strategies are most often observed before concrete counting‐on or counting‐on strategies and the concrete counting‐on strategy is typically observed before counting‐on. Nevertheless, at this stage of our investigation, and despite the fact that this sequence order is not arbitrary and is theoretically justified, it is not possible to assert that one strategy directly stems from the preceding one. Such a conclusion could only be drawn from intervention studies in which one strategy is explicitly taught with the expectation that it will accelerate the emergence of the subsequent strategy. One attempt in this direction was made by Baroody (1987), who trained 17 children in the modeling strategy and observed the effect on the emergence of more advanced strategies. The results were inconclusive, but, as acknowledged by the author, this may have been due to the small sample size. Future studies with larger samples are needed to test whether mastery of one strategy facilitates the acquisition of the next in the developmental sequence. More generally, regarding the teaching of specific finger‐counting strategies, it is possible that, for at least some of the children we observed, these strategies were not self‐discovered, as we have implicitly assumed throughout this discussion, but were explicitly taught by teachers or parents at home. In this context, counting‐on is the strategy most likely to have been explicitly taught, because it is generally considered the most efficient strategy by educators and is sometimes recommended for practice (e.g., Education Endowment Foundation [EEF], 2023). Future studies should therefore investigate the extent to which children's use of counting‐on or other strategies at a given point in development stems from their use of previous strategies or occurs independently as a result of explicit teaching.

Before concluding, there are two points to consider when reflecting on our results. First, it is possible that some critical observations had been missed during the course of the study. Indeed, because testing sessions were spaced six months apart, it is possible that some strategies were adopted and subsequently abandoned between two assessment points without being observed. More frequent testing, every three months or even monthly, might have reduced this issue, but the possibility of missing observations can never be entirely ruled out. Moreover, the fact that a dominant pattern of behaviors emerged across testing points indicates that, at the very least, our design was sufficiently fine‐grained to capture a meaningful developmental trajectory of children's finger strategy use prior to mental addition. Second, we reported that children in our sample came from middle to higher socioeconomical backgrounds and the transferability of our conclusion to children from lower backgrounds will be necessary in the future. In fact, even at the scale of this study, the robustness of our conclusions is interesting to investigate across schools. A complementary analysis of our results revealed that there was no overall effect of finger use across schools (χ2(2, *N* = 1253) = 1.539, *p* = 0.453). However, at the final testing point of our study, children from the city with the lowest socioeconomic status among the three cities in which we conducted our investigation showed more frequent finger use than children from the other two cities (χ2(2, *N* = 165) = 5.827, *p* = 0.054). This is consistent with the pattern observed by Jordan et al. (2008), who found that children from lower‐SES backgrounds rely on finger counting for a longer period than children from higher‐SES backgrounds. Nonetheless, such subtle differential patterns in our results encourage further research in diverse cultural contexts, including across different countries, to extend and further assess the generalizability of our conclusions.

## Author Contributions

**Marie Krenger** and **Catherine Thevenot** designed the study. Marie Krenger conducted the observations in schools and coded children's behaviors. Marie Krenger and Catherine Thevenot wrote the Introduction, Method, and Discussion sections of the manuscript. Marie Krenger and **Jeanne Bagnoud** analyzed the results and wrote the Results section. Catherine Thevenot supervised the work.

## Funding

The authors have nothing to report.

## Conflicts of Interest

The authors declare no conflicts of interest.

## Data Availability

The data necessary to reproduce the analyses presented here are publicly accessible on OSF: Krenger, M., Bagnoud, J., & Thevenot, C. (2025). *Finger counting strategies* [Data set]. Open Science Framework. https://osf.io/543yr/overview?view_only=5937fa7b3412495f9849e60c629c1368. Sharing codes or materials do not apply to the present study. The analyses presented here were not preregistered.

## Endnotes

1 These means also include children who were still using their fingers at the final testing point of our observations, which implies that the duration of finger use may be underestimated. Restricting the analysis to children who abandoned finger use during the study period (N = 70) would avoid this issue, but may bias the results toward more advanced children by excluding those who had not yet internalized the procedure. Nevertheless, whatever the choice made to conduct these analyses, the results are similar. Indeed, among these 70 children, the 25 who began with modeling used their fingers for an average of 2.2 sessions; the 10 who began with concrete counting‐on averaged 4.0 sessions; and the 5 who began with counting‐on averaged 2.0 sessions. The 2 children who began with both concrete counting‐on and counting‐on strategies averaged 3.0 sessions. The 15 children who started with both modeling and concrete counting‐on averaged 3.1 sessions, and the 13 children who started with only “other” finger strategies used their fingers for an average of 2.3 sessions.

2 This was evidenced both by the number of children using this strategy and by the frequency of its use. The results for these two variables (i.e., number of children using a strategy and frequency of use) were always consistent, indicating that the observed tendencies were not driven by a small number of children relying heavily on specific strategies. Consequently, in the remainder of this discussion, we use the expression “most commonly used” to refer jointly to these two types of observations.

3 The external measure of children's arithmetic abilities was derived from their scores at T7 on the Woodcock–Johnson Math Fluency task, in which as many written addition and subtraction problems as possible have to be solved in three minutes. Our results showed that children who followed a pathway different from the one hypothesized to best lead to the internalization of addition procedures (n = 26) did not perform significantly worse (M = 24.7 problems solved correctly) than the other children (n = 95, M = 28.3), t(119) = 1.67, p = 0.098, d = 0.22.

## Appendix A

### A.1

Number of children who had or had not internalized the finger counting strategy depending on their strategy repertoire developmental pathway, and comparison with Baroody's model of strategy development (1987).

**Table:**

	*N* of children			
Strategy combination developement	who had internalized	who had not internalized	Total	Order of strategy emergence
CC > CO		2	2	CC > CO
CC > CO = CC	1		1	CC > CO
CO = CC > CO		1	1	CC = CO
M > CO	6	12	18	M > CO
M > M = CC	2	2	4	M > CC
M > M = CC > CC		1	1	M > CC
M > M = CC > M	1	3	4	M > CC
M > M = CO > CO	1	1	2	M > CO
M > CC > CO		2	2	M > CC > CO
M > CC > M = CO = CC > CO	1		1	M > CC > CO
M > CO = CC	1	1	2	M > CC = CO
M > CO = CC > CO	1	2	3	M > CC > CO
M > M = CC > CC > CO = CC		1	1	M > CC > CO
M > M = CC > CO	1	2	3	M > CC > CO
M > M = CC > M = CO		1	1	M > CC > CO
M > M = CC > M = CO = CC > CO		1	1	M > CC > CO
M = CC > CC > CO	1		1	M = CC > CO
M = CC > CC > M = CC > CO		1	1	M = CC > CO
M = CC > CO	2	2	4	M = CC > CO
M = CC > CO = CC	1		1	M = CC > CO
M = CC > CO = CC > CO		1	1	M = CC > CO
M = CC > M = CO > CO	1	1	2	M = CC > CO
M = CC > M = CO = CC	1	1	2	M = CC > CO
M = CO = CC > CO	1	1	2	M = CC = CO
CC > CO = CC > CC	1		1	CC > CO
CC > M > M = CC	1		1	CC > M
CC > M = CC > M = CO = CC > M > M = CO		1	1	CC > M > CO
CC > M = CO > M = CO = CC > CO = CC > CO	1		1	CC > M = CO
CO = CC > CC > CO		1	1	CO = CC
CO = CC > CC > CO = CC > CO	1		1	CO = CC
M > CO = CC > M > CO > M = CO = CC		1	1	M > CO = CC
M > M = CC > M > CO = CC > M		1	1	M > CC > CO
M > M = CO = CC > M > CO		1	1	M > CC = CO
M = CC > CC > M = CC > M > M = CO > CO		1	1	M = CC > CO
M = CC > CO > CO = CC		1	1	M = CC > CO
M = CC > M > CO > CC		1	1	M = CC > CO
M = CC > M > M = CC	1		1	M = CC
M = CC > M > M = CC > M = CO = CC > CO		1	1	M = CC > CO
M = CC > M = CO > CC	1		1	M = CC > CO
M = CC > M = CO > M = CO = CC		1	1	M = CC > CO
M = CC > M	3		3	M = CC
M = CC > M > CO		3	3	M = CC > CO
CO > M = CC		1	1	CO > M = CC
CO > M	1		1	CO > M
CO > M = CO > CO		1	1	CO > M
M > CO > M = CC > CO		1	1	M > CO > CC
CC > CO = CC > CO	1		1	CC > CO
CC > M		2	2	CC > M
CC > M > CC > CO		1	1	CC > M > CO
CC > M > CO	2	3	5	CC > M > CO
CC > M > M = CC > CO		1	1	CC > M > CO
CC > M = CC	3		3	CC > M
CC > M = CC > CO		1	1	CC > M > CO
CC > M = CC > M	1		1	CC > M
CC > M = CC > M = CO = CC > CO		1	1	CC > M > CO
CO = CC > M > CO		1	1	CO = CC > M
CO = CC > M = CC		1	1	CO = CC > M
CO = CC > M = CO	1		1	CO = CC > M
CO = CC > M = CO > CO		1	1	CO = CC > M
M = CC	4		4	M = CC
CO	6	1	7	CO
M	13	4	17	M
Other only	7		7	Other only
TOTAL	70	72	142	

***Note***: M refers to the modeling strategy, CC to the concrete counting‐on strategy, and CO to the counting‐on strategy. *Other* refers to the exclusive use of undetermined finger‐based strategies and this category is reported only when children relied solely on this strategy, and not as part of any broader repertoire or developmental pathway.

## Appendix B Percentages of strategy used by finger users on each problem across testing points.

### B.1

**Table:**

	T1					T2					T3					T4					T5					T6					T7				
	Mental	Modeling	Concrete counting‐on	Counting‐on	Other	Mental	Modeling	Concrete counting‐on	Counting‐on	Other	Mental	Modeling	Concrete counting‐on	Counting‐on	Other	Mental	Modeling	Concrete counting‐on	Counting‐on	Other	Mental	Modeling	Concrete counting‐on	Counting‐on	Other	Mental	Modeling	Concrete counting‐on	Counting‐on	Other	Mental	Modeling	Concrete counting‐on	Counting‐on	Other
**Set1**	**56**	**15**	**11**	**1**	**17**	**54**	**22**	**13**	**2**	**9**	**58**	**28**	**7**	**1**	**6**	**67**	**22**	**5**	**2**	**4**	**73**	**19**	**2**	**2**	**4**	**85**	**9**	**2**	**2**	**2**	**91**	**3**	**1**	**3**	**1**
**Non‐tie**	**50**	**18**	**13**	**1**	**17**	**44**	**27**	**14**	**3**	**11**	**42**	**39**	**10**	**2**	**8**	**50**	**33**	**7**	**3**	**7**	**56**	**29**	**4**	**4**	**7**	**73**	**15**	**4**	**3**	**4**	**84**	**5**	**1**	**6**	**3**
1 + 2	61	12	9	0	18	66	17	9	2	6	63	28	4	1	4	74	20	3	0	4	86	11	1	0	2	94	4	0	0	2	98	1	0	0	1
1 + 5	55	9	18	0	18	38	28	15	4	15	44	39	8	0	8	56	29	7	2	5	66	27	3	3	2	89	7	2	0	1	96	4	0	0	0
2 + 3	48	30	12	0	9	36	32	19	0	13	41	37	11	0	11	49	31	6	4	9	57	24	6	1	12	79	6	4	2	9	86	2	1	6	4
3 + 4	45	18	12	3	21	45	26	13	4	13	31	47	13	3	7	37	41	12	4	7	35	39	5	7	15	52	30	5	7	5	69	11	2	12	5
4 + 5	42	21	15	3	18	34	34	17	4	11	31	43	13	4	9	33	46	9	4	8	35	46	5	8	6	52	29	7	7	4	69	9	4	14	5
**Tie**	**61**	**13**	**9**	**0**	**17**	**64**	**17**	**12**	**0**	**6**	**75**	**17**	**5**	**0**	**3**	**83**	**11**	**3**	**0**	**2**	**90**	**8**	**1**	**1**	**0**	**96**	**2**	**1**	**0**	**0**	**99**	**1**	**0**	**0**	**0**
1 + 1	76	9	3	0	12	83	6	4	0	6	91	7	0	0	3	95	3	2	0	1	96	4	0	0	0	100	0	0	0	0	100	0	0	0	0
2 + 2	73	12	3	0	12	83	6	6	0	4	80	12	4	0	4	89	6	0	0	4	96	4	0	0	0	100	0	0	0	0	99	1	0	0	0
3 + 3	42	21	12	0	24	45	28	15	0	13	69	23	7	1	0	78	14	5	1	2	83	12	2	1	2	91	4	4	0	1	98	1	0	1	0
4 + 4	64	12	9	0	15	53	26	15	2	4	60	25	11	0	4	75	17	4	0	4	84	13	3	0	0	91	6	1	0	1	98	1	0	1	0
5 + 5	52	9	18	0	21	57	17	21	0	4	73	17	3	0	7	80	13	5	1	1	88	9	1	2	0	98	2	0	0	0	100	0	0	0	0
**Set 2**	**29**	**44**	**15**	**3**	**8**	**24**	**43**	**27**	**3**	**4**	**31**	**31**	**22**	**5**	**11**	**35**	**30**	**18**	**8**	**9**	**47**	**23**	**12**	**8**	**10**	**56**	**11**	**6**	**16**	**12**	**66**	**6**	**1**	**22**	**5**
**Comp.10**	**36**	**39**	**17**	**3**	**6**	**27**	**42**	**24**	**1**	**5**	**34**	**30**	**20**	**3**	**12**	**40**	**28**	**17**	**7**	**8**	**51**	**23**	**9**	**7**	**10**	**70**	**9**	**3**	**11**	**8**	**81**	**5**	**0**	**12**	**2**
1 + 9	33	44	22	0	0	43	39	17	0	0	48	26	19	0	7	60	23	11	3	3	70	10	9	1	9	95	1	0	0	4	98	2	0	0	0
2 + 8	56	22	11	0	11	27	27	32	0	14	43	26	17	2	13	35	27	22	3	13	60	19	10	2	9	77	8	4	4	8	88	4	0	6	2
3 + 7	33	33	22	0	11	30	39	26	0	4	24	33	22	4	17	35	34	17	6	8	42	31	8	8	10	59	11	4	15	10	78	5	0	16	1
4 + 6	22	56	11	11	0	9	61	22	4	4	22	35	24	7	11	29	29	17	16	10	33	31	9	15	11	48	14	4	24	10	62	7	1	26	4
**Others**	**22**	**50**	**14**	**3**	**11**	**20**	**44**	**29**	**4**	**2**	**29**	**31**	**23**	**7**	**10**	**31**	**32**	**19**	**10**	**9**	**42**	**23**	**15**	**10**	**9**	**41**	**13**	**8**	**22**	**16**	**51**	**6**	**2**	**32**	**8**
2 + 6	33	56	11	0	0	22	48	26	0	4	30	35	20	6	9	35	37	18	6	4	61	23	8	3	5	61	16	4	10	9	78	5	2	11	4
3 + 8	33	44	11	0	11	27	36	32	5	0	33	28	22	6	11	36	29	16	8	11	50	21	13	10	6	59	8	5	13	15	68	6	0	22	4
4 + 9	11	67	22	0	0	14	50	32	5	0	28	30	24	9	9	29	29	19	13	9	36	21	21	12	10	29	13	10	30	18	36	7	1	42	14
5 + 7	11	33	11	11	33	17	43	26	9	4	24	33	26	7	9	24	31	22	12	12	23	28	20	14	15	16	14	14	34	22	22	7	5	53	12

## Appendix C Percentages of strategies used and percentages of correct responses (acc.) at each testing point in Set 1 by level 1 and level 2 finger users, and in Set 2 by Level 2 finger users.

### C.1

**Table:**

			T1		T2		T3		T4		T5		T6		T7^a^	
Skill level	Set	Strategy	4½ y‐o		5 y‐o		5½ y‐o		6 y‐o		6½ y‐o		7 y‐o		7½ y‐o	
			Use (%)	Acc.(%)	Use (%)	Acc.(%)	Use (%)	Acc.(%)	Use (%)	Acc.(%)	Use (%)	Acc.(%)	Use (%)	Acc.(%)	Use (%)	Acc.(%)
Skill level 1	Set 1	Modeling	7	53	10	44	33	59	11	89	13	67	23	43	—	—
		Concrete counting‐on	12	38	20	41	9	37	7	42	7	38	10	100	—	—
		Counting‐on	0	—	1	100	0	—	2	67	4	40	0	_	—	—
		Other finger strategies	21	12	15	25	11	26	11	32	3	25	7	0	—	—
		Mental	60	22	54	32	47	36	69	53	73	65	60	78	—	—
Skill level 2	Set 1	Modeling	38	94	34	91	26	89	24	90	19	94	8	88	3	76
		Concrete counting‐on	9	75	6	93	7	89	5	90	2	79	2	81	1	100
		Counting‐on	2	100	3	100	1	100	2	100	2	95	2	86	3	93
		Other finger strategies	6	60	3	67	4	79	3	87	4	82	2	88	2	92
		Mental	46	88	55	88	63	92	66	95	73	96	86	97	91	99
	Set 2	Modeling	44	38	43	28	31	24	30	42	23	51	11	73	6	86
		Concrete counting‐on	15	27	27	35	22	38	18	43	12	41	6	63	1	63
		Counting‐on	3	100	3	100	5	73	8	88	8	89	16	85	22	92
		Other finger strategy	8	0	4	43	11	40	9	59	10	57	12	83	5	88
		Mental	29	48	23	56	31	47	35	56	47	68	56	89	66	92

*Note*: ^a^At T7, there were no Level 1 finger user, thus this column is left empty.
